# Phosphorylation at Ser 727 Increases STAT3 Interaction with PKC*ε* Regulating Neuron–Glia Crosstalk via IL-6-Mediated Hyperalgesia In Vivo and In Vitro

**DOI:** 10.1155/2022/2782080

**Published:** 2022-01-28

**Authors:** Xiongjuan Li, Biqiang Zhou, Han Yang, Xinping Yang, Zhao Zhao, Zhenglong Pan, Xinran Liao, Wenling Jian, Yuqiang Liu, Han Lu, Qingsheng Xue, Yan Luo, Buwei Yu, Huansen Huang, Daqing Ma, Zhiheng Liu

**Affiliations:** ^1^Department of Anesthesiology, Shenzhen Second People's Hospital, The First Affiliated Hospital of Shenzhen University, Health Science Center, Shenzhen 518035, China; ^2^Department of Geriatric & Spinal Pain Multi-Department Treatment, Shenzhen Second People's Hospital, The First Affiliated Hospital of Shenzhen University, Health Science Center, Shenzhen 518035, China; ^3^Department of Anesthesiology, Ruijin Hospital Affiliated to Shanghai Jiaotong University, Shanghai 200025, China; ^4^Department of Anesthesiology, Second Affiliated Hospital of Guangzhou Medical University, No. 250 Changgangxi Road, Guangzhou 510260, China; ^5^Division of Anaesthetics, Pain Medicine and Intensive Care, Department of Surgery and Cancer, Faculty of Medicine, Imperial College London, Chelsea & Westminster Hospital, London, UK

## Abstract

**Methods:**

A rat hyperalgesia model was induced using an intraplantar injection of Freund's complete adjuvant (FCA) or an intrathecal injection of IL-6. Mechanical allodynia was evaluated using von Frey filament tests after intrathecal injections of T-5224 (c-Fos/AP-1 inhibitor), minocycline (Mino, a specific microglia inhibitor), L-2-aminoadipic acid (LAA, an astroglial toxin), PKC*ε* inhibitor peptide, APTSTAT3-9R (STAT3 inhibitor), or anti-IL-6 antibody. The c-Fos, GFAP, Iba-1, PKC*ε*, STAT3, pSTAT3^Tyr705^ and pSTAT3^Ser727^, and IL-6 expression at the spinal cord level was assessed by Western blot analysis. The interactive effects of PKC*ε* and STAT3 were determined using immunofluorescence staining and immunoprecipitation *in vivo* and *in vitro*. Interleukin-6 promoter activity was examined using luciferase assays.

**Results:**

T-5224, Mino, and LAA attenuated FCA- or IL-6-mediated inflammatory pain, with a decrease in c-Fos, GFAP, Iba-1, PKC*ε*, and IL-6 expression. PKC*ε* inhibitor peptide and APTSTAT3-9R reversed FCA-induced nociceptive behavior, while decreasing pSTAT3^Ser727^, IL-6, c-Fos, GFAP, and Iba-1 expression and PKC*ε* and STAT3 coexpression. Interleukin-6 promoter activity increased in the presence of PKC*ε* and STAT3. The interaction with PKC*ε* increased on phosphorylating STAT3 at Ser727 but not at Tyr705.

**Conclusion:**

STAT3 phosphorylation at Ser 727 and the interaction with PKC*ε* contribute to hyperalgesia *via* the IL-6-mediated signaling pathway, thus regulating neuron–glia crosstalk during inflammatory pain.

## 1. Introduction

Inflammatory mediators play important roles in pain development by interfering with nociceptive cellular signal transduction and transmission. The proinflammatory cytokine interleukin-6 (IL-6) is secreted by astrocytes and microglia in the central nervous system [1, 2]. It might play an important role in the development and maintenance of hyperalgesia in various pain models [3]. The upregulation of IL-6 in the spinal cord causes mechanical hyperalgesia in rats and is associated with the nociceptive sensory process [4–6]. The dysregulation of IL-6 results in the production and release of several inflammatory mediators that may activate neurocytes and trigger neuropathic pain [3, 7]. The blockade of IL-6 signaling leads to substantial clinical improvement in inflammatory arthritis [8].

Protein kinase C (PKC) is an important family of intracellular signaling enzymes involved in central sensitization and pain transmission [9]. The PKC epsilon (PKC*ε*) isoform is associated with the initiation of hyperalgesia by regulating nociceptor excitability [10, 11]. Signal transducer and activator of transcription 3 (STAT3) modulates gene expression involved in promoting inflammatory pain. Notably, PKC*ε* interacts with STAT3 [12], which targets and activates the IL-6 gene to increase IL-6 production [13, 14]. However, whether the interaction between PKC*ε* and STAT3 affects IL-6-mediated neuron–glia activation and hyperalgesia remains unknown.

In this study, we sought to explore the interaction between PKC*ε*, STAT3, and IL-6 during inflammation-induced hyperalgesia. First, we investigated whether PKC*ε*, STAT3, and IL-6 directly participated in neurocyte (including neurons, astrocytes, and microglia) activation during inflammatory pain in rats. Then, we aimed to determine the roles of PKC*ε* and STAT3 in IL-6-induced hyperalgesia and neuron–glia crosstalk [15, 16] *in vitro*. Finally, we examined the interactions between PKC*ε* and STAT3 as well as their effects on IL-6 promoter activity *in vivo* and *in vitro*.

## 2. Materials and Methods

### 2.1. Animals

A local experimental animal committee approved the experimental protocol, which was implemented according to the guidelines of the Institutional Animal Care and Use Committee (number 2019-004). Adult (6- to 8-week-old) male Sprague–Dawley rats weighing 200–220 g (*n* = 130) were acclimated for 1 week under a 12 h light/dark cycle at 22°C ± 2°C and 55% ± 5% relative humidity and received food and water *ad libitum*.

### 2.2. Reagents and Administration

Reagents and antibodies were obtained from the following suppliers: c-Fos/AP-1 inhibitor T-5224, PKC*ε* inhibitor peptide, and STAT3 inhibitor APTSTAT3-9R (Apexbio Technology LLC., TX, USA); specific microglia inhibitor minocycline hydrochloride (Mino) and astroglial toxin L-2-aminoadipic acid (LAA; Sigma–Aldrich Corp., MO, USA); and rat IL-6 and anti-IL-6 antibodies (PeproTech Inc., NJ, USA and Abcam, Cambridge, UK, respectively). Anti-IL-6 and Mino were diluted with phosphate-buffered saline (PBS) and saline, respectively; the other agents were dissolved in 1% dimethyl sulfoxide (DMSO). The aforementioned chemicals (50 *μ*L) were injected intrathecally *via* the L5-6 lumbar interspace identified by the tail flick reflex [17] under 1%–3% isoflurane (Baxter, IL, USA) anesthesia delivered at an oxygen flow rate of 1 L/min.

### 2.3. Inflammatory Pain and IL-6-Induced Hyperalgesia Model

Freund's complete adjuvant (FCA; Sigma–Aldrich Corp.) consisting of heat-killed *Mycobacterium tuberculosis* (1 mg/mL) in paraffin oil (150 *μ*L) was injected into the plantar area of the right hind paws of the rats under 1%–3% isoflurane anesthesia delivered with oxygen at a flow rate of 1 L/min. The left hind paw was not injected. Physical signs (e.g., redness and swelling) and pain behavior were monitored for 24 h after FCA injection. A model of hyperalgesia was established using naïve rats given an intrathecal injection of IL-6 (20 ng/50 *μ*L) through the L5–L6 lumbar interspace identified by the tail-flick reflex 10 min before drug administration under isoflurane anesthesia.

### 2.4. Experiments In Vivo

**Figure 1:**
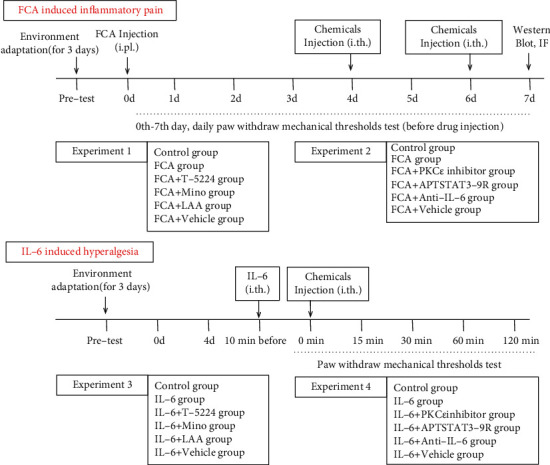
Schema of the experimental protocol. FCA: Freund complete adjuvant; IF: immunofluorescence; IL-6: interleukin-6; i.pl.: intraplantar; i.th.: intrathecal; LAA: L-2-aminoadipic acid; Mino: minocycline; PKC*ε*: protein kinase C epsilon.

Figure 1 shows the experimental protocol *in vivo.*

#### 2.4.1. Experiment 1

The rats were randomly assigned to the following groups (*n* = 6/group): untreated (control naïve rats), FCA, FCA + T-5224 500 *μ*g/50 *μ*L (T-5224), FCA + Mino 100 *μ*g/50 *μ*L (Mino), FCA + LAA 1 mg/50 *μ*L (LAA), and PBS 50 *μ*L (vehicle).

#### 2.4.2. Experiment 2

We investigated the roles of PKC*ε*, STAT3, and IL-6 in the inflammatory process by randomizing rats to the following groups (*n* = 6/group): untreated (control naïve rats), FCA, FCA + PKC*ε* inhibitor peptide 100 *μ*g/50 *μ*L (PKC*ε* inhibitor), FCA + APTSTAT3-9R 20 *μ*g/50 *μ*L (APTSTAT3-9R), FCA + anti-IL-6 antibody 100 ng/50 *μ*L (anti-IL-6), and 1% DMSO 50 *μ*L (vehicle).

#### 2.4.3. Experiment 3

We evaluated the effects of neuron-glial activity inhibitors on IL-6-induced hyperalgesia by randomizing rats to the following groups (*n* = 6/group): untreated (control naïve rats), IL-6, IL-6 + T-5224 500 *μ*g/50 *μ*L (T-5224), IL-6 + Mino 100 *μ*g/50 *μ*L (Mino), IL-6 + LAA 1 mg/50 *μ*L (LAA), and 1% DMSO 50 *μ*L (vehicle). All inhibitors were injected intrathecally 10 min before IL-6 (20 ng/50 *μ*L).

#### 2.4.4. Experiment 4

We assessed the effects of PKC*ε* and STAT3 on IL-6-induced hyperalgesia by randomizing rats to the following groups (*n* = 6/group): untreated (control naïve rats), IL-6, IL-6 + PKC*ε* inhibitor 100 *μ*g/50 *μ*L, IL-6 + APTSTAT3-9R 20 *μ*g/50 *μ*L, IL-6 + anti-IL-6 antibody 100 ng/50 *μ*L, and PBS 50 *μ*L (vehicle). All drugs were injected intrathecally 10 min before IL-6 (20 ng/50 *μ*L).

The paw withdrawal mechanical threshold (PWMT) was measured daily after the intraplantar injection of FCA (Figure 1). All agents described earlier were injected intrathecally on days 4 and 6 after the intraplantar injection of FCA. The spinal cords were harvested on day 7. In the IL-6-induced hyperalgesia model, the pain thresholds after chemical administration were assessed as the PWMT up to 120 min after IL-6 administration.

### 2.5. Von Frey Filament Tests

We measured PWMT using von Frey filament tests (Stoelting Co., IL, USA) using the up-and-down method [18]. The rats were habituated to a wire mesh platform for at least 1 h/d for 3 days before starting experiments. All groups (*n* = 6 each) were tested daily before drug application to determine baseline levels. Briefly, the positive and negative data were tabulated as follows: *X* = withdrawal and 0 = no withdrawal. The 50% response threshold was interpolated using the following formula: 50%g threshold = (10^(*xf* + *kδ*)^)/10,000, where *xf* is the last value (in log units) of the von Frey filament test, *k* is the tabular value for positive/negative responses, and *δ* is the mean difference (in log units) between stimuli [18]. The PWMT was defined as the means of six animals before and after chemical injections. Areas under receiver operator characteristic curves (AUC) were calculated to determine the effects of the injected chemicals.

### 2.6. Western Blot Analysis

The rats were euthanized using isoflurane anesthesia after the aforementioned tests. The lumbar segments (L3–L5) of whole spinal cords (*n* = 6/group) were homogenized and centrifuged at 16,000 g and 4°C for 15 min. Equal amounts of protein (50 *μ*g) in supernatants were denatured, resolved by sodium dodecyl-sulfate polyacrylamide gel electrophoresis (SDS-PAGE, 5% stacking gel, and 10%–12% separating gel), and then electrotransferred onto polyvinylidene difluoride (PVDF) membranes (Millipore, Massachusetts, USA). Nonspecific protein binding on the membranes was blocked with 5% nonfat milk in tri-buffered saline and Tween 20 (TBST) for 2 h at room temperature and then incubated with the primary antibodies: rabbit anti-c-Fos, anti-Iba-1, anti-PKC*ε*, anti-STAT3 (1 : 1000; Affinity Biosciences, OH, USA), anti-IL-6 (1 : 1000; Abcam, MA, USA), anti-GFAP, and anti-GAPDH (1 : 1000; Cell Signaling Technology, MA, USA). The blots were washed with TBST and then probed with secondary HRP goat anti-rabbit IgG (1 : 10,000) in 5% nonfat milk in TBST for 1 h at room temperature. The proteins of interest on the blots were visualized using an ECL reagent (Affinity Biosciences) and photographed using an X-ray film. The protein band intensity was quantified using the ImageJ software (National Institutes of Health, MD, USA).

### 2.7. Immunofluorescence Staining

After transcardial perfusion 7 days after FCA administration with 4% paraformaldehyde, the spinal cords were removed from the rats, postfixed, and dehydrated. Transverse frozen sections (10 *μ*m) prepared from OCT-embedded tissues were incubated overnight with rabbit polyclonal anti-PKC*ε* (Abcam) and mouse monoclonal anti-STAT3 (Cell Signaling Technology). The proteins in the tissues were visualized using a DMIL LED scanning microscope (Leica Microsystems GmbH, Wetzlar, Germany). Primary or secondary antibodies were omitted to ensure staining specificity. The data from three to four sections per rat (*n* = 6/group) were analyzed.

### 2.8. Immunoprecipitation Assays

Naïve rats were euthanized under isoflurane anesthesia. The lumbar segments L3–L5 from whole spinal cords were ultrasonicated in ice-cold RIPA buffer (Beyotime, Shanghai, China). The supernatant after low-speed centrifugation was diluted with lysis buffer and adjusted to 2 mg/mL. The cell suspensions passed through agarose resin served as controls. The sonicates were incubated overnight at 4°C with PKC*ε* and IgG primary antibody (5 *μ*L) to form immune complexes that were captured with fresh elution buffer and then incubated at 95°C for 6–8 min in RIPA buffer. The mixture was centrifuged, and then, endogenous PKC*ε*/STAT3 immune complexes in the supernatant were analyzed by Western blot analysis.

### 2.9. Cell Culture and Transfection

HEK293 cells (Shanghai Institutes for Biological Sciences, Shanghai, China) were cultured in Dulbecco's modified Eagle's medium (DMEM) containing 10% fetal bovine serum and 1% penicillin/streptomycin (Gibco; Thermo Fisher Scientific Inc., Waltham, MA USA) at 37°C in a 5% CO_2_ incubator (Thermo Fisher Scientific Inc.). Various constructs were transfected into HEK293 cells using Lipofectamine 2000 (Invitrogen; Thermo Fisher Scientific Inc.) as described by the manufacturer.

Full-length PKC*ε* and STAT3 were, respectively, cloned into pEGFP-C1 (Clontech Laboratories Inc., Mountain View, CA, USA) and pECMV-3X Flag-N (Biofeng, Beijing, China), then transfected into HEK293 cells. Two phosphorylation sites were mutated using point mutation technology to construct phenylalanine and alanine mutants STAT3^Y705F^ and STAT3^Y727A^, respectively, which mimic dephosphorylated STAT3. The aspartic acid mutants STAT3^Y705D^ and STAT3 mimic phosphorylated STAT3.

Extracts of transfected cells were quantified using BCA assay kits (Beyotime). Supernatants were diluted with lysis buffer and adjusted to 2 mg/mL of protein; then, 500 *μ*L portions were incubated overnight with 20 *μ*L of anti-Flag magnetic beads (Beyotime) at 4°C. Immune complexes with magnetic beads were washed three times with lysis buffer, precipitated, then eluted from the beads by boiling with 30 *μ*L of SDS-PAGE loading buffer for 6–8 min. The eluate was centrifuged; then, proteins in the supernatant were analyzed by western blotting.

### 2.10. IL-6 Promoter Activity

The IL-6 promoter region (-1500 to +19) was amplified by PCR and ligated into the pGL3 Basic vector (Biofeng). Full-length PKC*ε* and STAT3 were cloned into pRL-null cells (Biofeng), then transfected into cultured HEK293 cells. The pGL3-IL-6 promoter (0.5 *μ*g), pRL-null (Renilla, 0.5 *μ*g), pRL-STAT3 (1 *μ*g), and pRL-PKC*ε* (1 *μ*g) expression vectors were cotransfected overnight into HEK293 cells. The transfected cells were incubated with 1 *μ*g/mL lipopolysaccharide (LPS) for 12 h, and then, IL-6 promoter activity was assayed using dual luciferase kits (Jikai Gene Chemical Technology Co., Ltd., Shanghai, China).

### 2.11. Statistical Analysis

All data were presented as means ± standard deviation (SD) and analyzed by one-way analysis of variance (ANOVA) followed by the Bonferroni tests using SPSS 19.0 (IBM Corp., NY, USA). The graphs were constructed using the GraphPad Prism 7.0 software (GraphPad Software Inc., CA, USA). The statistical significance was set at *P* < 0.05.

## 3. Results

### 3.1. Inhibiting Activated Neurocytes Alleviated Inflammatory Pain and IL-6-Induced Hyperalgesia

**Figure 2:**
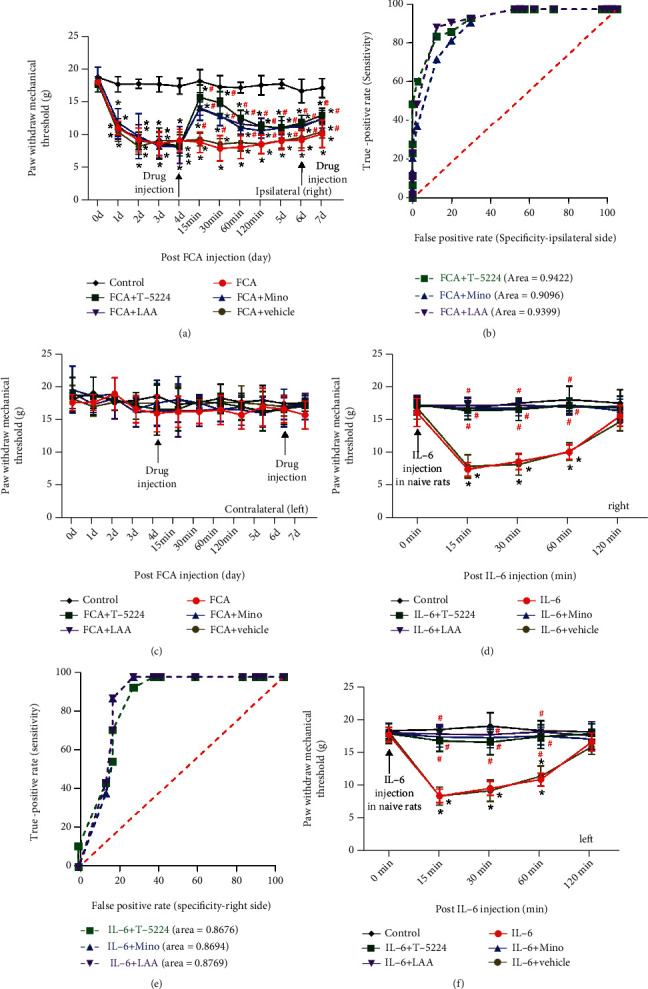
T-5224, Mino, and LAA ameliorated mechanical allodynia, inflammatory pain, and IL-6 induced hyperalgesia in rats. (a) Line plots indicate the effects of T-5224 (c-Fos/AP-1 inhibitor, 500 *μ*g/50 *μ*L), minocycline (Mino, 100 *μ*g/50 *μ*L), and L-2-aminoadipic acid (LAA, 1 mg/50 *μ*L) on ipsilateral paw withdrawal mechanical thresholds (PWMTs) in FCA-induced inflammatory pain. ^∗^*P* < 0.05 versus control. ^#^*P* < 0.05 versus FCA; one-way ANOVA with Bonferroni tests. (b) ROC curves and AUC of T-5224, Mino, and LAA on the ipsilateral side of FCA-treated rats. (c) Intrathecal injection of T-5224, Mino, and LAA did not alter FCA-induced mechanical nociception on the contralateral noninflamed side (*P* > 0.05). (d, f) Interleukin-6 (20 ng/50 *μ*L) microinjection reversed T-5224-induced mechanical hyperalgesia (500 *μ*g/50 *μ*L), Mino (100 *μ*g/50 *μ*L), and LAA (1 mg/50 *μ*L) in the right and left hind paws of naïve rats; ^∗^*P* < 0.05 versus control. ^#^*P* < 0.05 versus IL-6, one-way ANOVA with Bonferroni tests. (e) ROC curves and AUC for T-5224, Mino, and LAA in the right hind paws of naïve rats. Data are shown as means ± SD (*n* = 6). AUC: area under receiver operator characteristic curves; ROC: receiver operator characteristic curve.

Two injections each of T-5224, Mino, and LAA, respectively, inhibited the activation of neurons, astrocytes, and microglia cells and generated immediate and prolonged anti-FCA-induced inflammatory pain in the model (Figure 2(a)). The AUC of T-5224, Mino, and LAA in the ipsilateral side paw of the FCA-induced inflammatory pain model was, respectively, 0.94 ± 0.02, 0.91 ± 0.03, and 0.94 ± 0.02 (Figure 2(b)). These agents did not affect the mechanical threshold in the paw that was not injected (Figure 2(c)).

A single intrathecal injection of IL-6 (20 ng/50 *μ*L) evoked transient, but significant, mechanical allodynia in the right hind paws of naïve rats. This was similar to the FCA-induced mechanical hypersensitivity and was reversed by T-5224, Mino, and LAA (Figure 2(d)). The AUC of T-5224, Mino, and LAA was 0.87 ± 0.05, 0.87 ± 0.05, and 0.88 ± 0.05, respectively, in the right hind paws of the rats under IL-6-mediated hyperalgesia (Figure 2(e)). The analgesic effects of T-5224, Mino, and LAA were similar in the left hind paw under IL-6-induced hyperalgesia (Figure 2(f)).

### 3.2. T-5224, Mino, and LAA Decreased the Expression of c-Fos, GFAP, Iba-1, PKC*ε*, and IL-6

**Figure 3:**
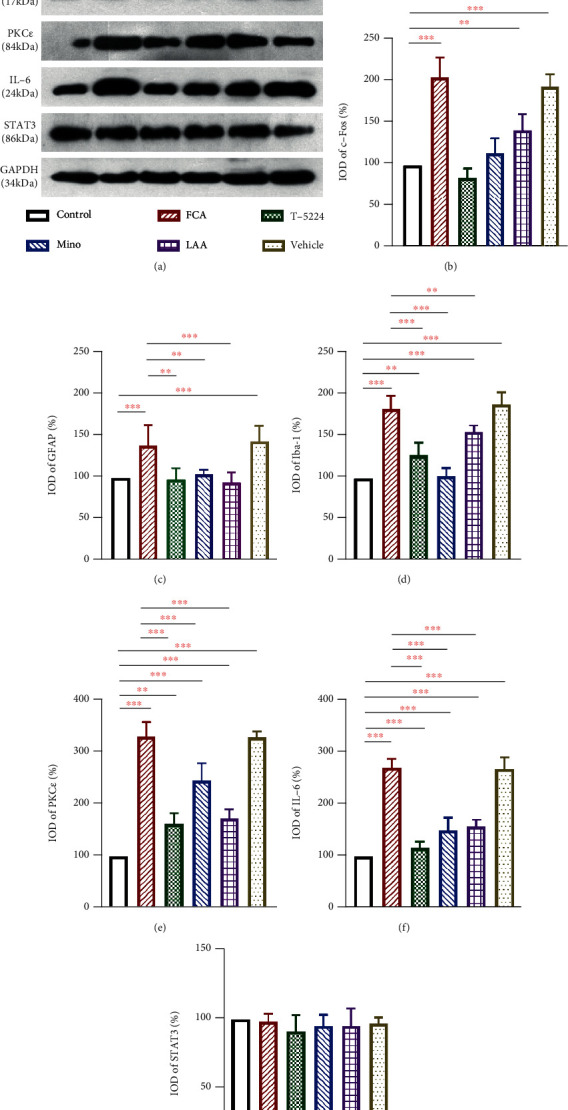
Western blot analysis of the proteins related to activated neurocytes during inflammatory pain. Examples (a) and mean values (b–g) of c-Fos, GFAP, Iba-1, PKC*ε*, STAT3, and IL-6 proteins in the spinal cord. Intrathecally injected T-5224 (c-Fos/AP-1 inhibitor, 500 *μ*g/50 *μ*L), minocycline (Mino, 100 *μ*g/50 *μ*L), and L-2-aminoadipic acid (LAA, 1 mg/50 *μ*L) reduced c-Fos, GFAP, Iba-1, PKC*ε*, and IL-6 protein levels in FCA-treated rats, whereas STAT3 expression did not change between groups (*P* > 0.05). Data were normalized against GAPDH and are expressed as ratios (%) of control. Data are shown as means ± SD (*n* = 4–5). ^∗^*P* < 0.05, ^∗∗^*P* < 0.01; ^∗∗∗^*P* < 0.001, one-way ANOVA with Bonferroni tests.

T-5224, Mino, and LAA significantly decreased the expression of c-Fos, GFAP, and Iba-1, which were, respectively, the FCA-induced markers of neurons, astrocyte, and microglia activation in the spinal cord (Figures 3(a)–3(d)). The expression of PKC*ε* and IL-6 in the spinal cord was also upregulated in FCA-treated rats (Figures 3(e) and 3(f)), whereas that of STAT3 did not differ among the groups (Figure 3(g)), indicating that the effects of T-5224, Mino, and LAA were exerted through the PKC*ε* and IL-6 pathways, but not via the STAT3 signaling pathway.

### 3.3. Roles of PKC*ε*, STAT3, and IL-6 in Inflammatory Pain and IL-6-Induced Hyperalgesia

**Figure 4:**
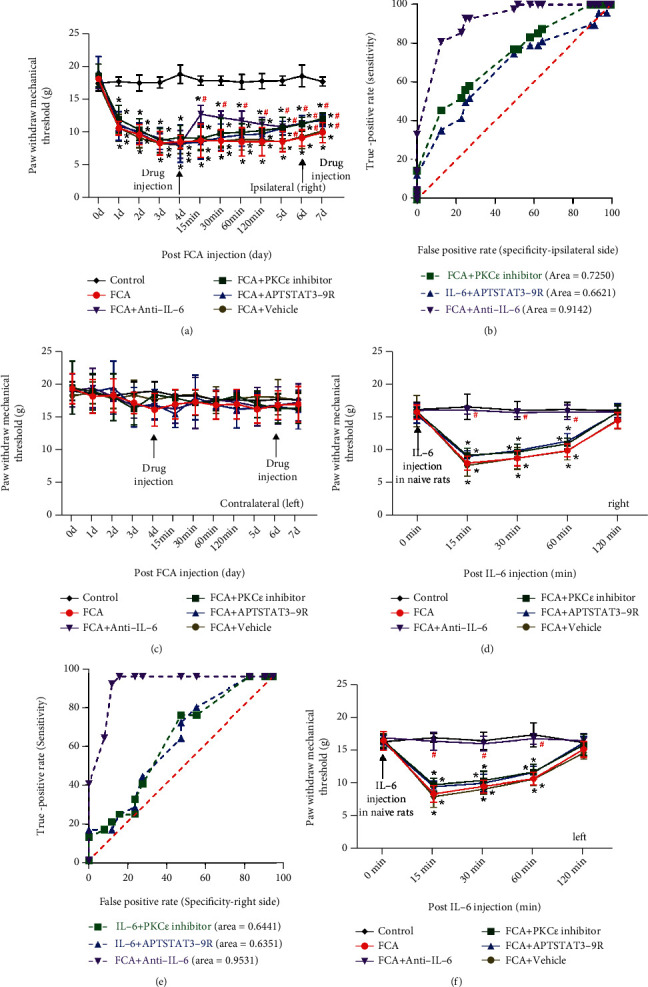
Evaluation of paw withdrawal mechanical threshold (PWMT) in rats with inflammatory pain and IL-6-induced hyperalgesia after the administration of PKC*ε* inhibitor peptide, APTSTAT3-9R (STAT3 inhibitor), or anti-IL-6 antibody. (a) Anti-IL-6 antibody (100 ng/50 *μ*L) rapidly attenuated FCA-induced pain for several days on the ipsilateral inflamed side. PKC*ε* inhibitor peptide (100 *μ*g/50 *μ*L) and APTSTAT3-9R (20 *μ*g/50 *μ*L) exerted antinociceptive effects 24 h after drug injection. ^∗^*P* < 0.05 versus control. ^#^*P* < 0.05 versus FCA, one-way ANOVA followed by Bonferroni tests. (b) ROC curves and AUC of PKC*ε* inhibitor peptide, APTSTAT3-9R, and anti-IL-6 on the ipsilateral side of FCA-treated rats. (c) None of the PKC*ε* inhibitor peptide, APTSTAT3-9R, and anti-IL-6 affected contralateral mechanical paw withdrawal thresholds in rats with FCA-induced inflammatory pain. (d, f) Interleukin-6 (20 ng/50 *μ*L) microinjected 10 min after naïve rats were injected with PKC*ε* inhibitor peptide (100 *μ*g/50 *μ*L) or APTSTAT3-9R (20 *μ*g/50 *μ*L) did not affect IL-6-induced mechanical hyperalgesia, whereas anti-IL-6 antibody (100 ng/50 *μ*L) did. ^∗^*P* < 0.05 versus control. ^#^*P* < 0.05 versus IL-6, one-way ANOVA followed by Bonferroni tests. (e) ROC curves and AUC of PKC*ε* inhibitor peptide, APTSTAT3-9R, and anti-IL-6 in the right hind paws of naïve rats. Data are shown as means ± SD (*n* = 6). AUC: area under receiver operating characteristic curve; ROC: receiver operator characteristics curve.

We blocked the corresponding cellular signaling pathways using PKC*ε* inhibitor peptide, APTSTAT3-9R, and anti-IL-6 antibodies to determine the roles of PKC*ε*, STAT3, and IL-6 in the pain process. Anti-IL-6 significantly increased mechanical threshold inflammatory pain on the ipsilateral, but not the contralateral, hind paw from the start of drug injection for up to 7 days, whereas PKC*ε* inhibitor peptide and APTSTAT3-9R increased mechanical pain threshold from days 5–7 (Figure 4(a)). The AUC of PKC*ε* inhibitor peptide, APTSTAT3-9R, and anti-IL-6 antibody was 0.73 ± 0.05, 0.66 ± 0.06, and 0.91 ± 0.03 in the ipsilateral side hind paw of the FCA-induced inflammatory pain model, respectively (Figure 4(b)). The intrathecal administration of T-5224, Mino, and LAA did not affect the FCA-induced mechanical threshold in the contralateral noninflamed paw (Figure 4(c)).

When these three conditions were applied to rats with IL-6-induced hyperalgesia, only anti-IL-6 improved the hypersensitivity and response to pain in the right paw (Figure 4(d)). The AUC of the PKC*ε* inhibitor peptide, APTSTAT3-9R, and anti-IL-6 antibody was 0.64 ± 0.08, 0.64 ± 0.08, and 0.95 ± 0.03 in the right hind paw under IL-6-mediated hyperalgesia, respectively (Figure 4(e)). The analgesic effect of anti-IL-6 was similar in the left hind paw (Figure 4(f)).

### 3.4. PKC*ε* Inhibitor Peptide, APTSTAT3-9R, and Anti-IL-6 Antibody Decreased the Expression of IL-6, c-Fos, GFAP, and Iba-1

**Figure 5:**
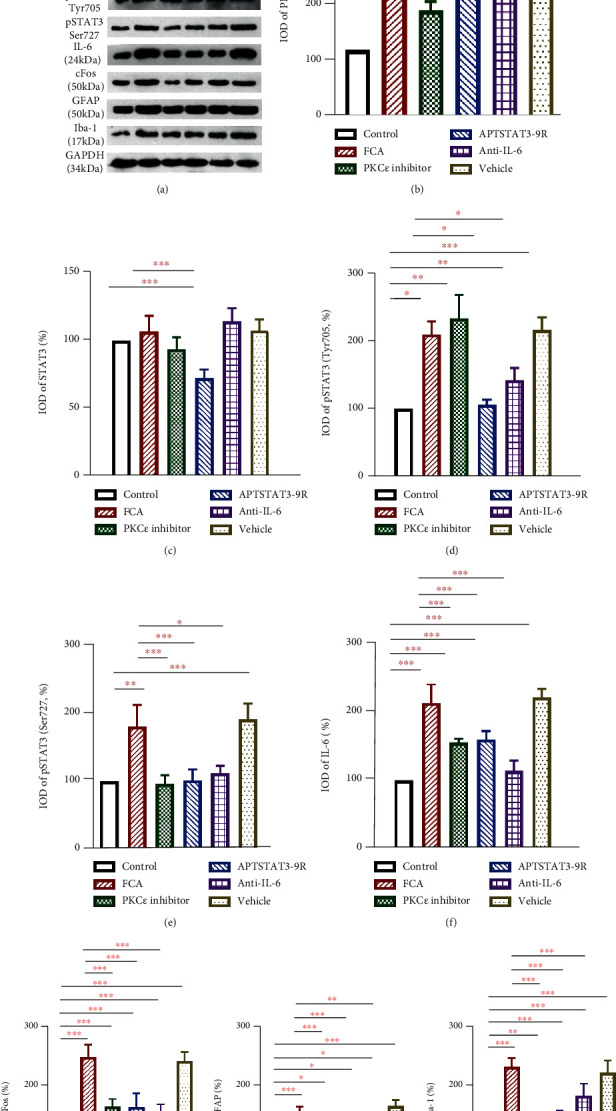
Expression of proteins related to activated neurocytes detected by Western blot analysis. (a–i) Protein expression of pSTAT3 (Ser727), IL-6, c-Fos, GFAP, and Iba-1 in the spinal cords of FCA-treated rats significant decreased (*P* < 0.05) after intrathecal injections of PKC*ε* inhibitor peptide (100 *μ*g/50 *μ*L), APTSTAT3-9R (20 *μ*g/50 *μ*L), and anti-IL-6 antibody (100 ng/50 *μ*L). Values were normalized against GAPDH and are expressed as ratios (%) of control values. Data are shown as means ± SD (*n* = 4–5). ^∗^*P* < 0.05, ^∗∗^*P* < 0.01, ^∗∗∗^*P* < 0.001; one-way ANOVA followed by Bonferroni tests.

The PKC*ε* inhibitor peptide reduced PKC*ε* expression, and APTSTAT3-9R downregulated the spinal level of STAT3 (Figures 5(a)–5(c)). The PKC*ε* inhibitor peptide, APTSTAT3-9R, and anti-IL-6 antibodies significantly decreased the spinal levels of phosphorylated STAT3^Ser727^ but not of STAT3^Try705^ and IL-6 (Figures 5(d)–5(f)). The expression of c-Fos, GFAP, and Iba-1 in the spinal cord was also decreased by the PKC*ε* inhibitor peptide, APTSTAT3-9R, and anti-IL-6 antibody in rats with FCA-induced inflammatory pain (Figures 5(g)–5(i)).

### 3.5. Phosphorylation of STAT3^Ser 727^ Increased STAT3 Interaction with PKC*ε*

**Figure 6:**
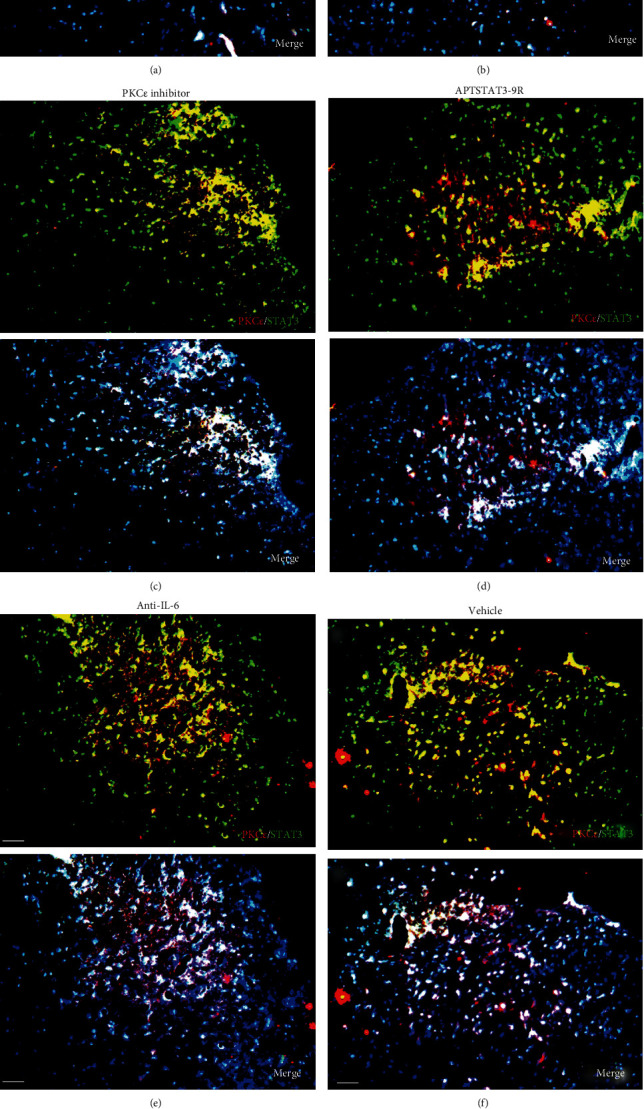
Detection of PKC*ε* and STAT3 coexpression *in vivo* and their immune complexes *in vitro*. (a–f) Immunofluorescence staining for PKC*ε* (red) and STAT3 (green) coexpression (yellow) and DAPI (blue in merged image) in spinal cord sections (a–e). Ratios of cells with immunoreactive PKC*ε*-/STAT3 among total cells. (f) Inhibitors of PKC*ε* and STAT3 significantly decreased the coexpression of PKC*ε*/STAT3 after APTSTAT3-9R administration. Bar = 40 *μ*m. Data are shown as means ± SD (*n* = 6). ^∗^*P* < 0.05, ^∗∗^*P* < 0.01, ^∗∗∗^*P* < 0.001; one-way ANOVA followed by Bonferroni tests.

The expression of PKC*ε*/STAT3 in the dorsal horn of the spinal cord was significant decreased by PKC*ε* or STAT3 inhibitor but not by anti-IL-6 antibody compared with that in the control and FCA groups (Figures 6(a)–6(f)).

**Figure 7:**
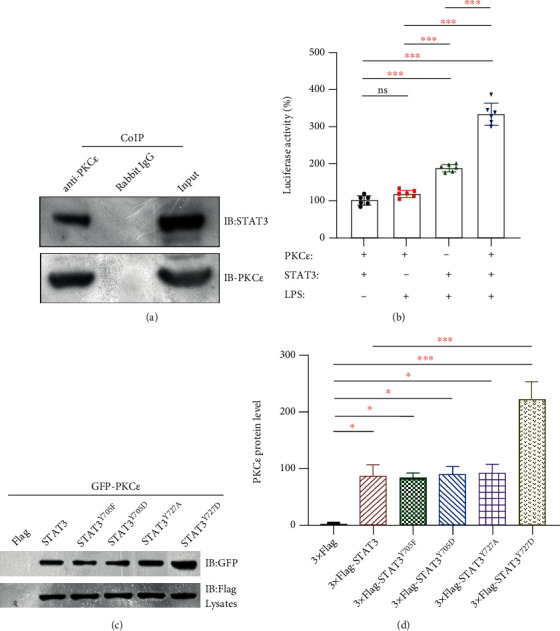
Ser727 of STAT3 increased its interaction with PKC*ε*. (a) Endogenous PKC*ε* was immunoprecipitated from cell lysates, and immune complexes and total cell lysates were analyzed by Western blot analysis with PKC*ε* and STAT3 antibodies. Endogenous immune PKC*ε*/STAT3 complexes were detected in the rat spinal cord tissues. (b) IL-6 promoter-firefly luciferase reporter plasmid (0.5 *μ*g), PKC*ε* (1 *μ*g), and STAT3 (1 *μ*g) were cotransfected overnight into HEK293 cells. The transfected cells were incubated with 1 *μ*g/mL lipopolysaccharide (LPS) for 12 h. Interleukin-6 promoter activity increased by STAT3 was further enhanced by PKC*ε* and STAT3, indicating that PKC*ε* improved the ability of STAT3 to bind to IL-6 promoter. Data are shown as means ± SD (*n* = 6). ^∗^*P* < 0.05, ^∗∗^*P* < 0.01, ^∗∗∗^*P* < 0.001; one-way ANOVA followed by Bonferroni tests. (c, d) HKE293 cells were transfected with GFP, GFP-PKC*ε*, Flag, Flag-STAT3, and phosphomimetic and dephosphomimetic mutants of STAT3, and then, immunoprecipitants were assayed. Protein complexes were detected using an anti-GFP antibody (c), and then, relative PKC*ε* binding to STAT3 was quantified (d). Data are presented as means ± SD (*n* = 3). ^∗^*P* < 0.05, ^∗∗^*P* < 0.01, ^∗∗∗^*P* < 0.001; one-way ANOVA followed by Bonferroni tests.

Endogenous PKC*ε*/STAT3 immunocomplexes in spinal cord tissues were assessed (Figure 7(a)). After incubating HEK293 cells with lipopolysaccharide (LPS), STAT3 increased IL-6 promoter activity, which was also enhanced in the presence of PKC*ε* (Figure 7(b)). The STAT3^Y727D^ phosphomimetic mutant had more affinity for PKC*ε*, whereas the other mutants generated results similar to those of wild-type STAT3, indicating that the phosphorylation at Ser727 increased the ability of STAT3 to bind to PKC*ε* (Figures 7(c) and 7(d)).

## 4. Discussion

Our results showed that FCA-induced inflammatory pain and IL-6-induced hyperalgesia were alleviated by inhibiting neurocyte activation or by anti-IL-6 therapy, indicating that IL-6 participated in the maintenance of inflammation-induced nociception. This study was novel in demonstrating that T-5224, Mino, and LAA inhibited FCA-induced inflammatory pain and IL-6-induced hyperalgesia, despite previous findings of the therapeutic effects of Mino against chronic bone cancer pain and chronic pain [19, 20].

The present study used an inflammatory pain model created by unilateral injections of FCA or intrathecal injections of IL-6 that induced hyperalgesia in rats. The mechanical paw withdrawal threshold was significantly reduced for up to 7 days by FCA and up to 60 min by IL-6. An intraplantar injection of FCA induced central sensitization and increased the levels of pain mediators, including IL-6, peripherally and centrally, whereas the intrathecal injection of IL-6 likely resulted in transient central sensitization due to a local increase in pain mediators per se [6, 21].

We found that T-5224, Mino, and LAA decreased the expression levels of c-Fos, GFAP, Iba-1, PKC*ε*, and IL-6 but did not alter STAT3 levels during the FCA-induced inflammatory process (Figure 3). Our results indicated that the inhibition of neurocyte activation reduced IL-6-mediated pain sensitivity, which was in line with previous findings of the crosstalk between activated neurocytes and IL-6-induced pain [3, 6, 16, 22, 23]. However, the analgesic effects of T-5224, Mino, and LAA were associated with other cytokines such as IL-1*β* and TNF-*α* [24], suggesting that activated neurocytes comprised a control mechanism of inflammatory pain [25].

**Figure 8:**
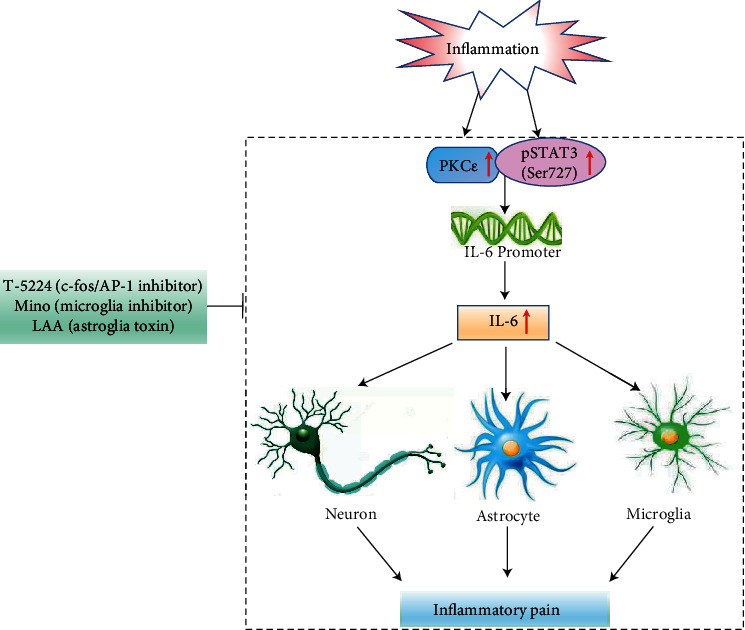
Interaction between PKC*ε* and STAT3^Ser727^ in nociceptive regulation during inflammatory pain. PKC*ε* and STAT3 engaged with IL-6-mediated neuronal and glial cell crosstalk during inflammatory nociceptive transmission. Under inflammatory conditions, upregulated PKC*ε* and STAT3^Ser727^ phosphorylation interacted in the spinal cord and subsequently increased IL-6 promoter activity that manifested as IL-6 cytokine-mediated inflammatory pain. T-5224, Mino, and LAA inhibited the PKC*ε*/STAT3/IL-6 signaling pathway and the activation of neurons, astrocytes, and glial cells during inflammatory pain development. IL-6: interleukin-6; LAA: L-2-aminoadipic acid; Mino: minocycline; PKC*ε*: protein kinase C epsilon; STAT3: signal transducer and activator of transcription 3.

We further examined the roles of PKC*ε*, STAT3, and IL-6 in FCA-induced inflammatory pain and IL-6-induced hyperalgesia. Anti-IL-6 immediately alleviated inflammatory pain for an extended period and reversed the hyperalgesic effects of IL-6 (Figure 4), suggesting that IL-6 was a potent pain mediator. In contrast, the PKC*ε* inhibitor peptide and the STAT3 inhibitor did not exert immediate analgesic effects but elicited a mild, sustained analgesic effect for 5–7 days at the end of the experimental period. This phenomenon was accompanied by a decrease in the pSTAT3^Ser727^ level but not in the levels of pSTAT3^Tyr705^, IL-6, c-Fos, GFAP, and Iba-1 in the spinal cord, indicating that the upstream effectors PKC*ε* and STAT3 regulated the formation of IL-6, thus mediating the activation of neurocytes (Figures 5 and 8). These findings were comparable with the association of PKC*ε* and STAT3 levels with the IL-6 level and neurocytes [26–30]. PKC*ε* and STAT3 regulated the production of IL-6, and pSTAT3^Ser727^ formed complexes with PKC*ε* and enhanced STAT3 localization to the IL-6 promoter, thus increasing IL-6 expression [31]. The decrease in the levels of pSTAT3 and IL-6 in cells incubated with APTSTAT3-9R and anti-IL-6 suggested that STAT3 was involved in a negative feedback loop in the IL-6-induced signaling pathway per se.

PKC*ε* and STAT3 interacted under physiological and pathological conditions [11, 31]. Our immunofluorescence results also revealed the coexpression of PKC*ε* and STAT3 in cells in the spinal cord (Figure 6), suggesting that PKC*ε* together with STAT3 contributed to IL-6 production and the activation of neurons, astrocytes, and microglia during inflammation. Alone, PKC*ε* did not affect the activity of the IL-6 promoter; but IL-6 promoter activity was increased more in the presence of both PKC*ε* and STAT3 than that in the presence of STAT3 alone under LPS stimulation (Figure 7), suggesting that PKC*ε* increased the ability of STAT3 to bind to the IL-6 promoter. The immunoprecipitation results *in vitro* showed that the pSTAT3^Ser727^ but not pSTAT3^Tyr705^ affected the interactions between PKC*ε* and STAT3 (Figure 7), suggesting that pSTAT3^Ser727^ regulated the formation of PKC*ε*/STAT3 complexes, thus influencing IL-6-mediated inflammatory pain.

Given the analgesic effect of T-5224, Mino, and LAA, they may serve as the potential therapeutic agents for inflammatory pain-related disease. This needs further discussion to understand the potential pharmacological characteristics of these compounds. T-5224 was first designed to inhibit the arthritis upstream of inflammatory cytokine and matrix metalloproteinase action [32]. It can be used in human articular chondrocytes, resulting in the inhibition of transactivation of downstream matrix metalloproteinases and inflammatory cytokines (including IL-6, IL-1*β*, and TNF-*α*) and effectively preventing cartilage destruction and osteophyte formation in an osteoarthritis-induced mouse model [33]. T-5224 was also found to be used in acute myeloid leukemia [34], mast cell [35], and triple negative breast cancer [36], which might serve as a synergistic therapeutic strategy for the clinical diseases; however, T-5224 is still in the preclinical stage. Hence, the data related to pharmacokinetics are lacking; more research and evidence are needed in the future. Mino is a semisynthetic tetracycline antibiotic with anti-inflammatory properties, which is used to treat multiple inflammatory diseases and could be safely applied in the clinical setting, such as Parkinson's disease [37] and neurodegenerative and psychiatric diseases [38] as well as the cerebral ischemia [39]. It is generally well tolerated, and skin-related complaints, nausea, and dizziness are the most common patient-reported side effects [40]. LAA, a selective astrocytic toxin, has been demonstrated to exert some regulatory effects on tibia fracture [41], myotubes [42], and retina [43], thus contributing to the fracture-induced nociceptive, cell autophagy in myotubes, and retinal vascular responses. It has not yet been clinically applied due to its unusual astroglial toxin, which may trigger locomotor network damage. How to reduce the toxicity of LAA to central and peripheral nerves to the minimum is worth exploring [44].

This study had some limitations. It focused only on IL-6-induced pain, and thus, its clinical relevance is debatable. However, early and delayed IL-6 elevation is associated with chronic neuropathic pain [45]. Interleukin-6 plays a key role in the chronic inflammation associated with rheumatoid arthritis (RA), and blocking IL-6 signaling is an important strategy in treating RA-associated diseases clinically [46]. Moreover, targeting IL-6 might be an option for treating other chronic inflammatory diseases [47]. Therefore, an in-depth understanding of how IL-6 induces cellular signaling that causes pain, and the development of new analgesic strategies associated with IL-6, have theoretical and clinical significance for pain management.

In this study, we found that the phosphorylation at Ser727 increased STAT3 interaction with PKC*ε*. This increased IL-6 promoter activity and upregulated IL-6 expression, thus enhancing neuron–glia activation during the development of inflammatory pain. In addition, the PKC*ε* inhibitor peptide and STAT3 inhibitor (APTSTAT3-9R) attenuated FCA-induced nociceptive behavior *via* IL-6 downregulation (Figure 8).

## 5. Conclusions

In summary, pSTAT3^Ser727^ interaction with PKC*ε* contributes to FCA-induced inflammatory pain and IL-6-mediated hyperalgesia *via* IL-6-modulating crosstalk among neurons, astrocytes, and microglia and their activation. The translational value of our findings warrants further investigation.

## Data Availability

The data used to support the findings of this study are available from the corresponding upon request.

## Abbreviations

APTSTAT3-9R: STAT3 inhibitor
AUC: Area under the receiver operating characteristic curve
DMEM: Dulbecco's modified Eagle's medium
FCA: Freund's complete adjuvant
GFAP: Glial fibrillary acidic protein
IF: Immunofluorescence
IL-6: Interleukin-6
LAA: L-2-Aminoadipic acid
LPS: Lipopolysaccharide
Mino: Minocycline
PKC*ε*: Protein kinase C epsilon
PWMT: Paw withdraw mechanical threshold
ROC: Receiver operator characteristics curve
STAT3: Signal transducer and activator of transcription 3.
