# Correction to “Phosphorylation at Ser 727 Increases STAT3 Interaction with PKCε Regulating Neuron–Glia Crosstalk via IL‐6‐Mediated Hyperalgesia In Vivo and In Vitro”

**DOI:** 10.1155/mi/9757469

**Published:** 2026-04-08

X. Li, B. Zhou, H. Yang, X. Yang, Z. Zhao, Z. Pan, X. Liao, W. Jian, Y. Liu, H. Lu, Q. Xue, Y. Luo, B. Yu, H. Huang, D. Ma, and Z. Liu, “Phosphorylation at Ser 727 Increases STAT3 Interaction with PKCε Regulating Neuron–Glia Crosstalk via IL‐6‐Mediated Hyperalgesia In Vivo and In Vitro,” *Mediators of Inflammation*, 2022, 2782080, https://doi.org/10.1155/2022/2782080.

In the article, there are errors in Figure 6, highlighted on PubPeer [1], in which duplications were introduced in multiple panels during the production process. The correct Figure 6 is below:

Figure 6Detection of PKCε and STAT3 coexpression in vivo and their immune complexes in vitro. (a–f) Immunofluorescence staining for PKCε (red) and STAT3 (green) coexpression (yellow) and DAPI (blue in merged image) in spinal cord sections (a–e). Ratios of cells with immunoreactive PKCε‐/STAT3 among total cells. (f) Inhibitors of PKCε and STAT3 significantly decreased the coexpression of PKCε/STAT3 after APTSTAT3‐9R administration. Bar = 40 μm. Data are shown as means ± SD (*n* = 6).  ^∗^ *p*  < 0.05,  ^∗∗^ *p*  < 0.01,  ^∗∗∗^ *p*  < 0.001; one‐way ANOVA followed by Bonferroni tests.

**Figure:**
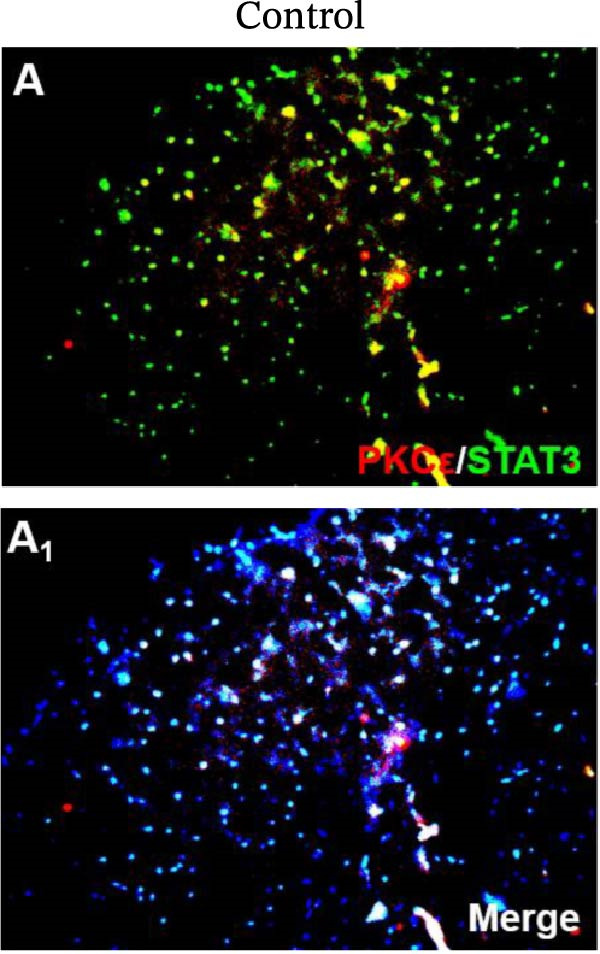
(a)

**Figure:**
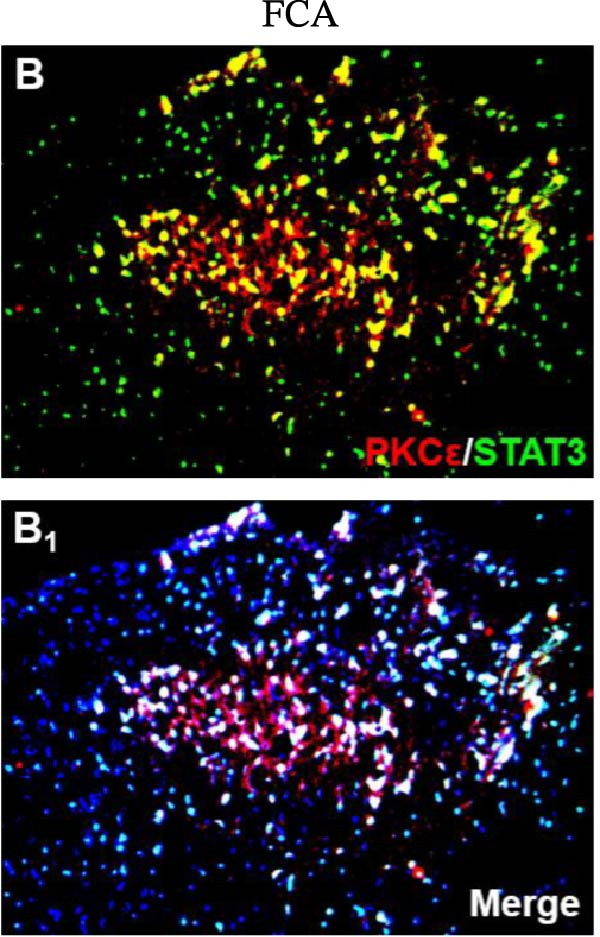
(b)

**Figure:**
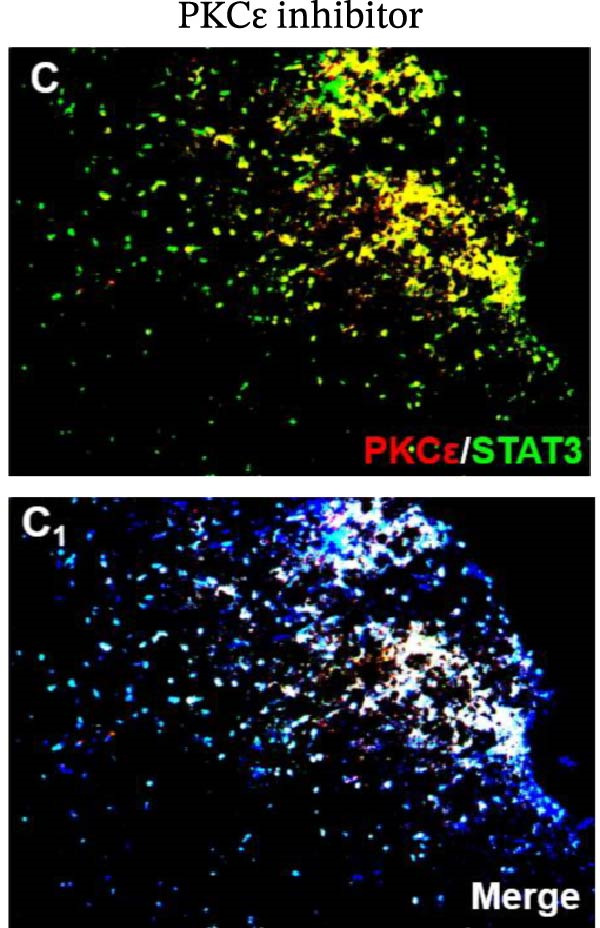
(c)

**Figure:**
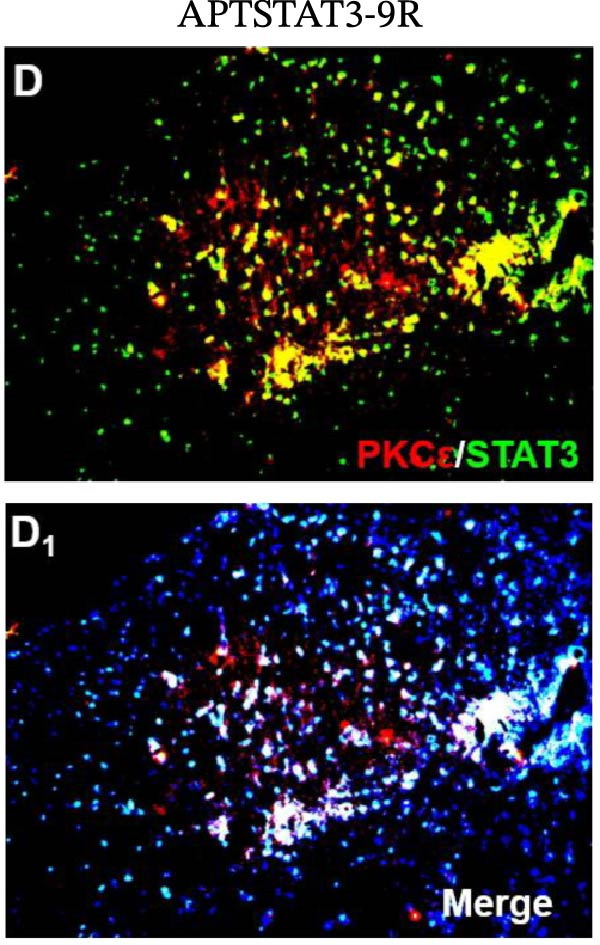
(d)

**Figure:**
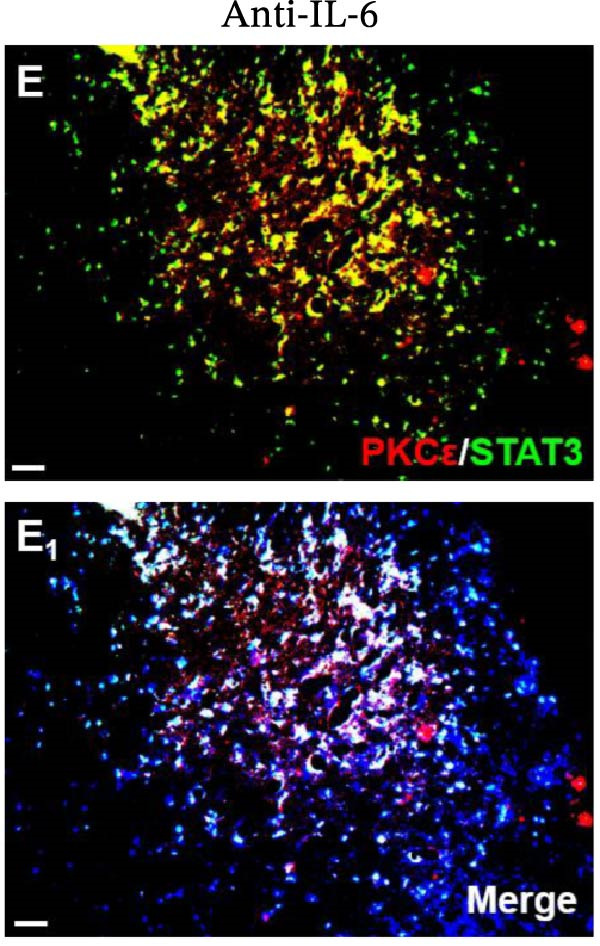
(e)

**Figure:**
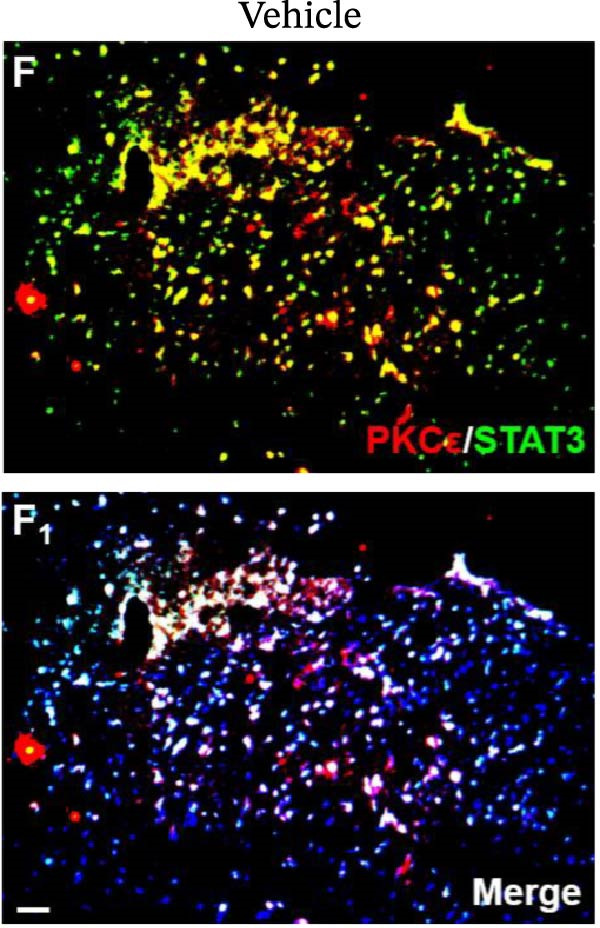
(f)

**Figure:**
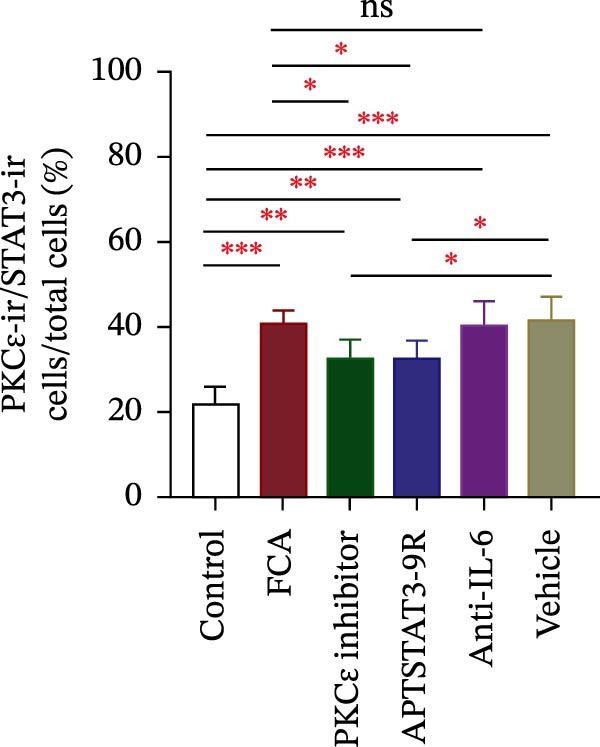
(g)

We apologize for these errors.
